# PSCC: Sensitive and Reliable Population-Scale Copy Number Variation Detection Method Based on Low Coverage Sequencing

**DOI:** 10.1371/journal.pone.0085096

**Published:** 2014-01-21

**Authors:** Xuchao Li, Shengpei Chen, Weiwei Xie, Ida Vogel, Kwong Wai Choy, Fang Chen, Rikke Christensen, Chunlei Zhang, Huijuan Ge, Haojun Jiang, Chang Yu, Fang Huang, Wei Wang, Hui Jiang, Xiuqing Zhang

**Affiliations:** 1 BGI-Shenzhen, Shenzhen, China; 2 Department of Clinical Genetics, Aarhus University Hospital, Aarhus, Denmark; 3 Department of Obstetrics and Gynaecology, The Chinese University of Hong Kong, Shatin, NT, Hong Kong; 4 State Key Laboratory of Bioelectronics, School of Biological Science and Medical Engineering, Southeast University, Nanjing, China; 5 Guangzhou Children's Social Welfare Home, Guangzhou, China; 6 Clinical laboratory of BGI Health, Shenzhen, China; 7 The Guangdong Enterprise Key Laboratory of Human Disease Genomics, BGI-Shenzhen, Shenzhen, China; The Scripps Research Institute, United States of America

## Abstract

**Background:**

Copy number variations (CNVs) represent an important type of genetic variation that deeply impact phenotypic polymorphisms and human diseases. The advent of high-throughput sequencing technologies provides an opportunity to revolutionize the discovery of CNVs and to explore their relationship with diseases. However, most of the existing methods depend on sequencing depth and show instability with low sequence coverage. In this study, using low coverage whole-genome sequencing (LCS) we have developed an effective population-scale CNV calling (PSCC) method.

**Methodology/Principal Findings:**

In our novel method, two-step correction was used to remove biases caused by local GC content and complex genomic characteristics. We chose a binary segmentation method to locate CNV segments and designed combined statistics tests to ensure the stable performance of the false positive control. The simulation data showed that our PSCC method could achieve 99.7%/100% and 98.6%/100% sensitivity and specificity for over 300 kb CNV calling in the condition of LCS (∼2×) and ultra LCS (∼0.2×), respectively. Finally, we applied this novel method to analyze 34 clinical samples with an average of 2× LCS. In the final results, all the 31 pathogenic CNVs identified by aCGH were successfully detected. In addition, the performance comparison revealed that our method had significant advantages over existing methods using ultra LCS.

**Conclusions/Significance:**

Our study showed that PSCC can sensitively and reliably detect CNVs using low coverage or even ultra-low coverage data through population-scale sequencing.

## Introduction

Copy number variations (CNV) are known to be an important component of structural variation in the human genome, resulting from a mixture of meiotic recombination, homology-directed and non homologous repair of double-strand breaks, and errors in replication [1]. CNVs contain duplication, deletion and multiallelic variation events of genetic material 1 kb or larger in size, and might have functional impact through gene expression and dosage [2], [3]. It has been reported that CNVs confer high risk for inherited diseases, complex diseases and cancer, such as autism spectrum disorders [4], systemic lupus erythematous [5] and neuroblastoma [6]. Common CNVs represented in more than 1% of the population are defined as copy number polymorphisms (CNP). These polymorphisms may contribute to phenotypic variations and differences in disease susceptibility across different ethnic groups [6], [7]. Therefore the detection and population-scale association analysis of CNVs is necessary for the study of migration and evolution, as well as for clinical diagnosis.

For the last 10 years, the Array Comparative Genomic Hybridization (aCGH) and Multiplex Ligation Probe Amplification (MLPA) methods have provided ample literature on the detection of CNVs [8], [9], [10]. Recently, massive parallel sequencing has begun to offer genome-scale detection of CNVs through high throughput, high-resolution methods. The Paired-End Read Mapping (PEM) strategy was the first sequencing-based strategy to detect CNVs, and is able to identify both insertions and deletions with a resolution at kilobase level by comparing the differences between the mapped read distance and the average library insert size, though it is unable to detect insertions larger than the average library insert size and the exact borders of the CNVs [11], [12]. Later, a split-read (SR) method was created to detect deletions and small insertions, but this method is restricted in the unique regions of the genome and cannot detect duplications with exact breakpoint resolution. Then the read counts (RC) method was developed to detect CNVs by comparing the reads aligned to a particular region and the expected value calculated according to the region's proportion in the whole genome; this method was first used in tumor samples [13], [14]. The read counts method can achieve a high resolution in a relatively low coverage sequencing, and some recently popular algorithms are based on it, including SegSeq [14], CNV-seq [15], CNAseg [16], ReadDepth [17] and rSW-seq [18]. However, most of these algorithms need one real or theoretical comparative genome, which induce an additional cost or unexpected fluctuations that prevent further large-scale association research and clinical applications.

Ideally, the number of reads located in a certain region is proportional to its copy number, which makes it possible to detect the copy number by counting directly counting the number of reads. However, the uniformity of read distribution is influenced by two main sources of bias: local GC bias and other multiplex-related bias. The GC bias has been well reported in previous papers [14], [19], [20]; moreover, there are multiplex reasons excepting GC content that could cause sequence bias, such as chromosomal structure [21]. In the case of multiplex-related bias, which is hard to observe and correct by individual/limited samples, it is necessary to recruit population-scale sequencing instead of using theoretical expectations or single comparative controls. With population-scale low-coverage whole genome sequencing (LCS), the sequence bias could be corrected to an acceptable level, which would be highly beneficial when creating new methods for CNV detection. Also, comprehensive approaches can be recruited to control the false positive rate in low coverage analysis with the use of population-scale data.

To achieve these goals, we have developed a population-scale CNV calling (PSCC) method, a new bioinformatics method to detect CNVs using population-scale LCS. PSCC consists of three modules, including a two-step correction procedure to remove the local GC content bias and multiplex-related bias, a binary segmentation method to locate the candidate CNV regions, and a combined statistics test to estimate the signal reliability and determine the CNV genotypes. To evaluate the performance of the PSCC method, we tested its sensitivity and specificity using ∼2× and ∼0.2× LCS data *in silico*, and then conducted detection of CNVs in 34 clinical samples.

## Methods

### Overview of PSCC algorithm

**Figure 1:**
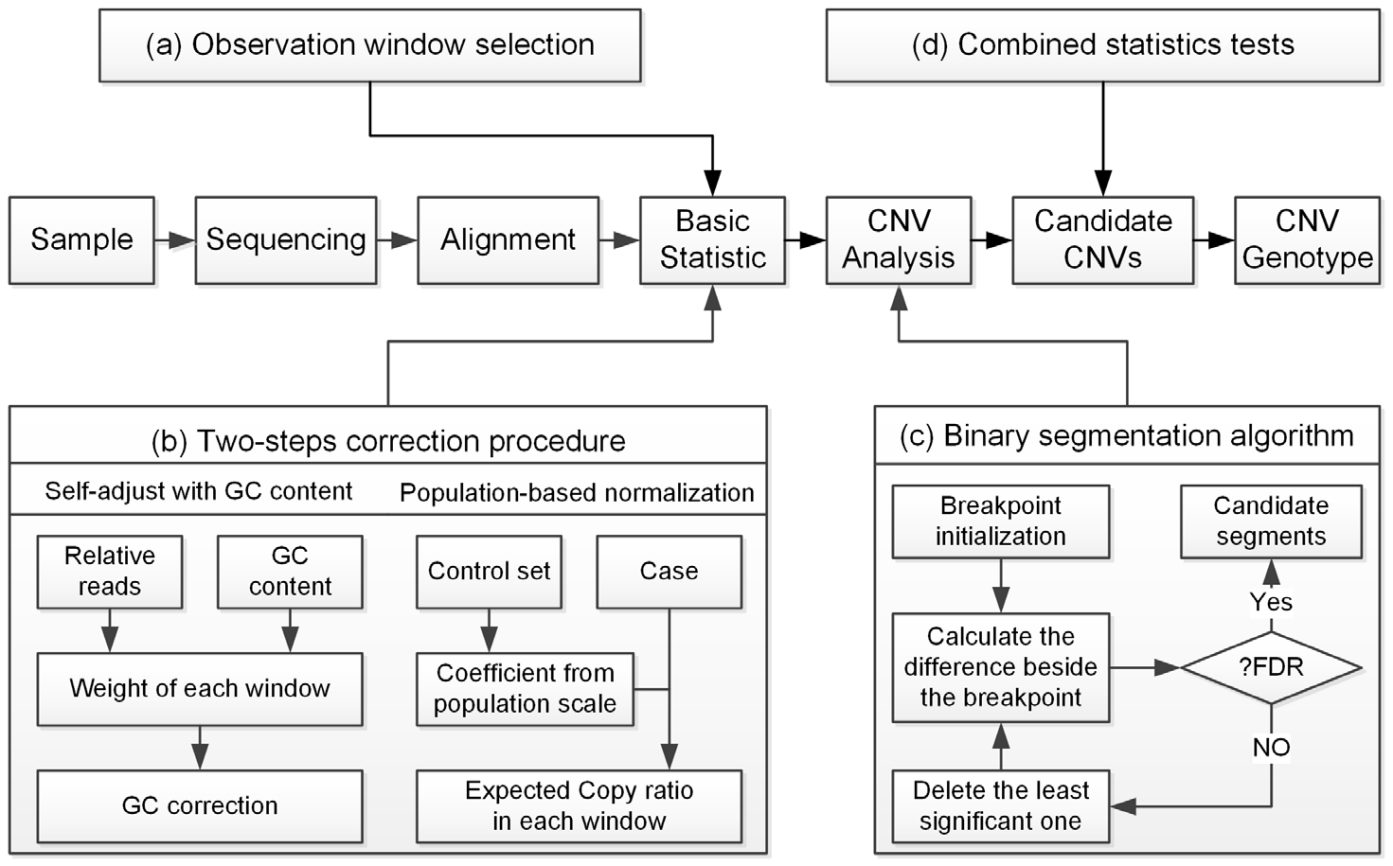
Bioinformatics pipeline of our copy number variation analysis strategy.

The aim of PSCC is to detect CNVs through population-scale sequencing using LCS data. For better CNV detection, we first recruited a two-step correction procedure to remove the local GC content bias and multiplex-related bias, then used a binary segmentation method to localize the CNV breakpoint, and finally utilized a combined statistics test to combat false positive controls. The implementation of PSCC mainly consists of the following steps (Figure 1):

- Observation window selection
- Two-step correction procedure
- Binary segmentation algorithm
- CNV genotype determination and combined statistics test

### Observation window selection

To take into account the various mapping reads and the sequencing reads of different regions of the human genome, we used adjustable sliding windows in the genome to calculate the statistic of RCs. The human reference genome was smashed into sliding simulated reads in the same paired-end/single-end type and read length as the case samples, and then remapped to the reference. Then the window sizes were adjusted to have the same expected RC, which is more comparable among windows in the correction process. Previous papers have reported that the optimal window size is inversely linked with the coverage, resulting in ∼30 bp bins for 100× coverage, ∼100 bp bins for 20–30× coverage, and ∼500 bp bins for 4–6× coverage [22]. Since the sequencing length was single-end 36 bp, it meant that an expected RC would range from 55 to 84 in each window. We estimated our optimum bin size with the actual sequencing depth according to this principle:
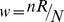
(1)


Where 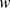 is the number of simulated reads that should be located in each window, 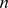 is the expected 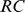 of the actual sample in each window, 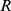 is the number of unique mapped reads in the simulated data, and 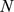 is the total sequencing reads.

### Two-step correction procedure

#### Bias correction with local GC content

In each window, the local GC content of each window is calculated as the average GC content of mapped reads. Strong correlation between RC and GC content has been reported in several studies [23], [24], [25]. The bias is suspected to be introduced during PCR in library preparation and cluster generation in the Illumina sequencing workflow [26]. In this study, our data indicates that the RC will be underrepresented in GC-poor and GC-rich regions, implying a significant GC bias. Here, we propose to adjust the RC by using the observed deviation in coverage for a given GC percentage [20]. In practice, for all the GC percentages we determine the deviation of coverage from the genome average and then correct each RC according to the following formula:
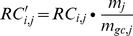
(2)


Where 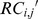 are read counts of the 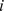-th window in sample 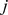, 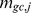 is the median 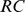 of windows that have the same GC content with 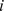-th window, and 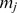 is the overall median of all the windows in sample 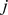.

#### Bias correction with population-based normalization

Irrespective of the GC content, there are other factors that influence the library preparation and cluster generation process such as complex genomic characteristics, and in the follow-up alignment the sequence homology and repeat structure seriously affect the mappability. So the RC becomes uniform in the single sample after the GC correction, but can still show significant bias in population-scale statistics. These biases should be similar in the same multisampling window due to the chromosome inherent attributes. Based on this assumption, we developed a population-based normalization procedure to eliminate these multiplex-related biases. The mean of each window in a control set is calculated to replace the expected value of single sample, while the standard deviation will be used to evaluate the polymorphism and to enact a false positive control in the subsequent procedures. This process is executed by means of the following formula:
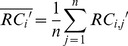
(3)

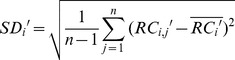
(4)

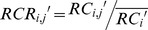
(5)


Where 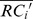 and 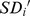 are the mean and standard deviation of corrected read counts of the 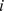-th window in all the 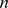 control samples. The 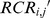 is the relative ratio of corrected read counts of the 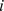-th window in sample 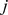.

Through this two-step correction, we can obtain an unbiased relative copy ratio (RCR) for each window. We also simultaneously obtain the deviation level in the population-based normalization process, which can be used to estimate the reliability of these regions that may not lead to a false positive.

### Binary segmentation algorithm

Based on the corrected RCR data, CNVs can be identified as a decrease or increase of the RCR across multiple consecutive windows. Before CNV genotype determination, we need to localize the segment breakpoints to identify the candidate CNV regions. The data that we obtain are mathematically very similar to the signal obtained from aCGH experiments, and the events in RCR data can be detected using the same algorithmic approaches that have been used for aCGH data [27]. At present, few statistical methods have been developed and tested for the detection of CNVs. Those methods in existence include circular binary segmentation algorithm (CBS) [28], shifting level model (SLM) [29], mean-shift algorithm (MSB) [22] and events significance testing (EWT) [20]. Here, we merge the adjacent windows with similar RCR into segments using a binary segmentation algorithm.

First, a set of candidate breakpoints is selected by calculating the significance of differences (p-value) of each window within the neighboring windows by a non-parameter test. The Wald-Wolfowitz runs test [30], which has a better performance in polymorphic conditions, is recruited here. A group of windows with minimal p-values were selected as ordered initialized candidate breakpoints.

Then an iterative algorithm is used to obtain the optimized candidate breakpoints set. In each loop, the lowest significant breakpoint (with maximum p-value) will be deleted and the p-values of neighboring windows will be refreshed. This procedure will be performed until all of the p-values are less than the genome-wide significance threshold. Thus, a set of candidate CNV regions are divided by these optimized breakpoints.

### CNV genotype determination and combined statistics tests

After segmentation, the CNV genotype of each segment must be determined to provide new insights for further research and clinical applications. With consideration regarding the data stability and sequence parallelism, we recruited two statistical tests to estimate the signal reliability and determine the CNV genotypes.

First, we evaluated the significance of each segment as a CNV using the U-test (alpha = 0.001); we will refer to this test as self-test (ST). After this evaluation, we utilized the Parallelism-test (PT) to estimate the reliability of each signal under the consideration of multiplex-related bias. The test statistics can be obtained from these formulas:
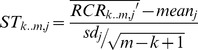
(6)

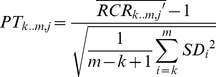
(7)


Where 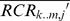
is the mean of the test region that contains windows ranging from 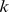 to 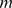, 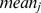 and 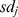 are the mean and standard deviation of sample 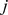, and 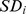 is the standard deviation of the 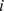-th window in control samples.

In PT, the CNVs in stable regions will show a significant *p-value*. Therefore, it can remove unreliable signals in unstable regions that are easily affected by the experimental process or chromosomal structures, such as tandem repeat. After ST and PT, we can classify CNVs into four situations.

- When ST and PT are both insignificant, the CNV genotype is consistent with the control set as normal.
- When ST and PT are both significant, it is a high-credible deletion/duplication.
- When ST is significant but PT is insignificant, it will be regarded as a false signal caused by instability of sequence or multiplex-related bias.
- When ST is insignificant but PT is significant, it is regarded as a false signal and shows an inappropriate sequence quality of this sample. A replication will be advised when t his kind of phenomenon happens a lot.

With the use of ST and PT, we effectively determined most CNV genotypes, and successfully filtered most of the false positive signals, resulting in a favourable sensitivity and a significant improvement in specificity.

## Results

### Performance of two-step bias correction

For the purpose of data observation and method development, samples from 90 normal Chinese individuals (CS) (data from the 1000 Genomes Project) and 34 Chinese individuals with Down Syndrome (DS) were recruited for the data observation. The CS samples were sequenced ∼5× and the DS samples were sequenced ∼1× in paired-end reads. (Table 1) Before further data analysis, we selected appropriate windows for this study. In order to keep the expected RCs, we defined the adjusted window using the simulated methods (described in Materials and Methods S1). Based on formula 1, we got a series of window sizes that had expected RCs of 25, 50, 100, 150, 250 and 500 in DS samples. To set up the appropriate expected RCs, RC distribution under different copy numbers (1, 2, 3) were presented (Figure S1 in Materials and Methods S1). Generally, the shared proportions of these three distributions decrease with more expected RCs in each window. In theory, with more expected RCs, our method will show an increased sensitivity and specificity but a decreased resolution. To obtain a balanced performance, we empirically used 150 as the expected RC in the following analysis.

**Table 1:** Date production of sample using in this study.

Samples	Numbers	Number of reads (M)	Total base (Gb)	Mapping rate (%)	Depth (X)	Coverage (%)
CS	90	170.91±25.65	17.09±2.57	89.28±1.86	5.27±0.74	96.28±2.87
DS	34	46.52±7.12	4.65±0.71	87.50±1.66	1.70±0.10	59.61±4.84
DC	34	116.38±16.09	5.70±0.79	94.37±0.27	2.22±0.21	74.86±5.14

**Figure 2:**
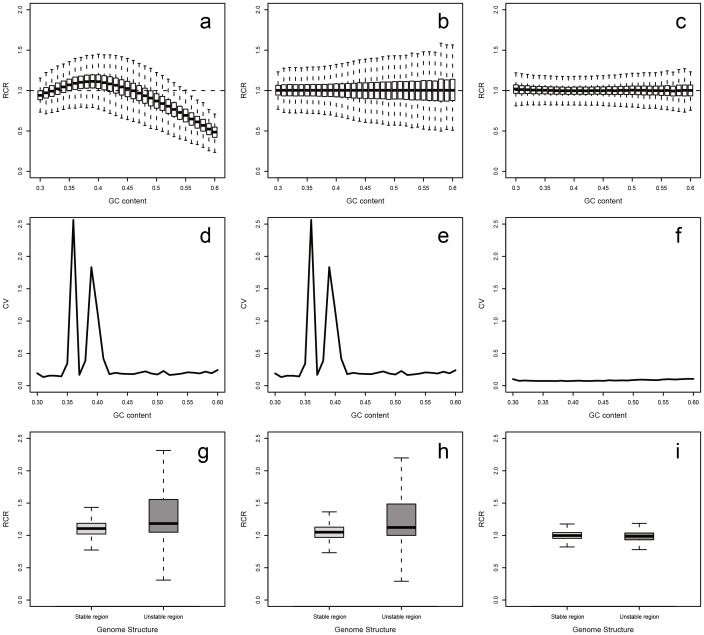
RCR distribution in correction process. These plots represent the correction effects of the two-step correction methods in an observed sample (CS-NA18632). (a, b, c) Performance in the RCR distribution [original (a), after correction step-1 (b), after correction step-2 (c)]. (d, e, f) Performance in coefficient of variation changement [original (d), after correction step-1 (e), after correction step-2 (f)]. (g, h, i) Performance of the RCR correction effect in the regions that have the similar GC content (i.e. 38∼42%) but different genome structure (the left box is the stable regions, the right box is the regions that close to centromeres, telomeres or N regions) [original (g), after correction step-1 (h), after correction step-2 (i)].

Under the RC strategy's assumption that the reads in any location of the genome are random, the RCs of each window should follow Poisson distribution. However, in practice the RC distribution showed a severe bias [20], [31]. In our CS samples, the RC was considerably under-represented in GC-poor (GC<35%) and GC-rich (GC >50%) regions (Figure 2a). Also, the coefficient of variation (CV) indicated significant sequence instability in these regions (Figure 2d). The existence of this GC bias may lead to an unsatisfied performance. To eliminate this GC bias, we applied the GC correction procedure to the RC, following formula 2 (Methods). After this process, most windows were centralized to a genome-wide level (RCR = 1), showing less bias between RCR and GC content (Figure 2b). In the case of CV, the amount had decreased to some degree; however, the CV of GC-poor and GC-rich regions was still at a considerably high level (Figure 2e). This instability will still influence the performance of CNV detection. Moreover, in terms of special structures, such as repeat regions, GC correction showed less effectiveness in stability improvement (Figure 2g, 2h). Therefore, it is necessary to correct these multiplex-related biases using population-scale sequencing.

In multiplex-related bias correction, we first calculated the expected RC of each window using 90 CS samples (formula 3, Methods). Afterwards, we normalized the corrected RC and obtained the RCR, following formula 5 (Methods). After multiplex-related bias correction, the distribution of RCR was more uniformly related to GC content, especially GC-rich regions (Figure 2c). The amount of CVs significantly decreased, showing a great improvement in data stability (Figure 2f). The RCRs of repeat regions were also more centralized, implying better data uniformity in special structures. In conclusion, we obtained relatively unbiased statistic data after the two-step correction procedure, which will benefit further CNVs detection processes.

### CNV detection power estimation *in silico*

When combined with the segmentation algorithm and CNVs genotype determination strategy (Methods), we can detect CNVs with a population-scale control set. For comprehensive evaluation of the performance of PSCC, we performed an intensive simulation based on synthetic data generated from RCRs of the 90 CS samples (‘Sample recruitment’ in Materials and Methods S1). In our simulation, the CNV size ranged from 20 kb to 10 Mb (Table S3 in Materials and Methods S1), and the sequence depth ranged from 0.2× to 20×. The sensitivity/specificity was estimated at call level in our simulation.

**Figure 3:**
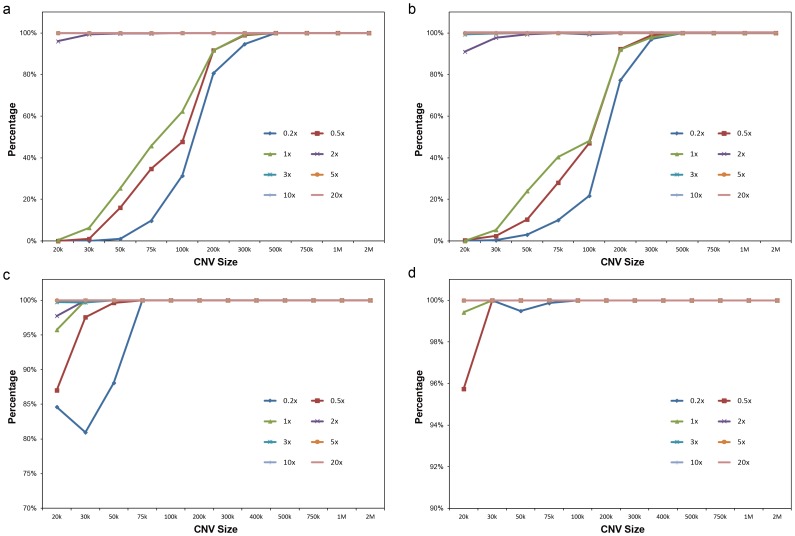
The sensitivity and specificity of PSCC in simulated CNVs detection process. In these plots, X-axis stands for the CNV size ranged from 0.2 to 20×, and Y-axis was the percentage. The sequencing depth are color-coded and distinguished by characters. (a, b) Sensitivity of simulated CNV detection [Duplication (a), Deletion (b)]; (c, d) Specificity of simulated CNV detection [Duplication (c), Deletion (d)].

Generally, the sensitivity increased with larger size and higher sequence depth (Figure 3 a, b). For the most part, CNVs over 100 kb could be detected with accuracy over 99.6% using 2× sequence data, while only 26.5% could be detected when we used 0.2× sequence data. The simulation indicated that it was able to detect large segment CNVs at ultra-low sequence depth. For instance, the sensitivity of deletion and duplication were 97% and 94% respectively when the sequence depth was 0.2× and the CNV size was larger than 300 kb. In the case of specificity, most of the false positive signals occurred at small sizes, and the results of deletion were more accurately detected than results of duplication. (Figure 3 c, d) For example, all the false deletions and duplications were smaller than 100 kb when the sequence depth was 0.2×. For the CNVs smaller than 20 kb, the specificity of deletion was 100% when the sequencing depth reached 2×, whereas duplications needed a depth of 5×.

### CNV calling in clinical samples

To estimate the performance of PSCC in actual practice, we recruited 34 Danish (DC) patients with clinical abnormal characterization in this study, which was approved by the Institutional Review Board of BGI. Written informed consent was obtained from all participants. 30 samples had been analyzed using a 180 k Agilent oligo nucleotide array-based comparative genomic hybridization (aCGH) platform prior to this study on an indication of developmental delay, and 4 older samples had been analysed with a Blue Gnome BAC array. CNVs detected by aCGH were executed a strict filter operation by existing databases (details in Materials and Methods S1). In 24 of these 34 clinical samples at least one pathogenic CNV had been identified in each sample, and in the other 10 samples, no obvious pathogenic CNV had been identified by aCGH analysis. Finally, a total of 31 pathogenic CNVs were certified, including 19 deletions (from 0.4 Mb to 18.3 Mb) and 12 duplications (from 0.4 Mb to 59 Mb) (details in Table S2 in Materials and Methods S1).

DNA was extracted directly from 34 peripheral blood samples and subsequent sequencing generated about 58 million paired-end 50-bp reads (i.e. about 2× coverage). All of the raw sequencing data had been submitted to NCBI SRA (http://www.ncbi.nlm.nih.gov/sra) and the Submission ID was SRA080273. The sequence reads were mapped to the reference genome (HG19, NCBI build 37) using SOAP2, and unique paired alignment reads were used for the following analysis (Table 1).

For CNV detection of these 34 DC samples, we successfully detected 47 CNVs, including 25 deletions and 22 duplications (Table 2). Among these 47 CNVs, 31 CNVs (19 deletions and 12 duplications) were consistent with the pathogenic CNVs in aCGH results (Table S2 in Materials and Methods S1). For the other 16 CNVs, 6 deletions ranged from 300 kb to 1.2 Mb and 10 duplications ranged from 300 kb to 700 kb. Of those CNVs, we chose 12 of them, including 4 deletions and 8 duplications, for a Real-time Quantitative PCR (qPCR) validation because of limited DNA amount and primer design difficulty for the left locus. (Table S4 in Materials and Methods S1) All these 12 CNVs were validated to be accurate by qPCR (Figure S2 in Materials and Methods S1). However, when we reviewed the benign CNVs in aCGH data that were defined as polymorphism in Database of Genomic Variants, 13 of these 16 CNVs were contained. (Table S2 in Materials and Methods S1) It illustrated that most of these CNVs maybe polymorphism in population, which has not been eliminated in the population-based normalization due to the insufficient control set of normal samples.

**Table 2:** Statistics of CNVs detection by PSCC for 34 clinical samples.

Variation Type	Size	Detected by PSCC	Consisted with pathogenic CNVs in aCGH	Consisted with benign CNVs in aCGH	Validation of qPCR^*^
Deletion	300 k∼1 M	7	2	4	3/3
	1 M∼5 M	11	10	-	1/1
	5 M∼10 M	4	4	-	-
	>10 M	3	3	-	-
Duplication	300 k∼1 M	12	2	9	8/8
	1 M∼5 M	3	3	-	-
	5 M∼10 M	3	3	-	-
	>10 M	4	4	-	-

Validation of qPCR, the results were expressed as A/B. A means the number of successful validated and B means the total number of validate loci.

### Performance comparison between different CNVs detection methods

To evaluate the overall performance of our methods, we also analysed these 34 clinical samples using available CNV detection methods, SegSeq and ReadDepth. In methodology, SegSeq and ReadDepth are significantly different from our method. SegSeq uses a comparative genomic strategy to decrease the experimental variance, while ReadDepth recruits a theoretical distribution to detect CNVs. Another important difference is that SegSeq and ReadDepth use relatively fixed thresholds to determine the CNV genotypes. To detect the CNVs, we executed SegSeq with suggested parameters (-W 400 –a 1000 –b 10), using the CS samples as control (Materials and Methods S1), and executed ReadDepth with the default parameters downloaded from the official website.

**Figure 4:**
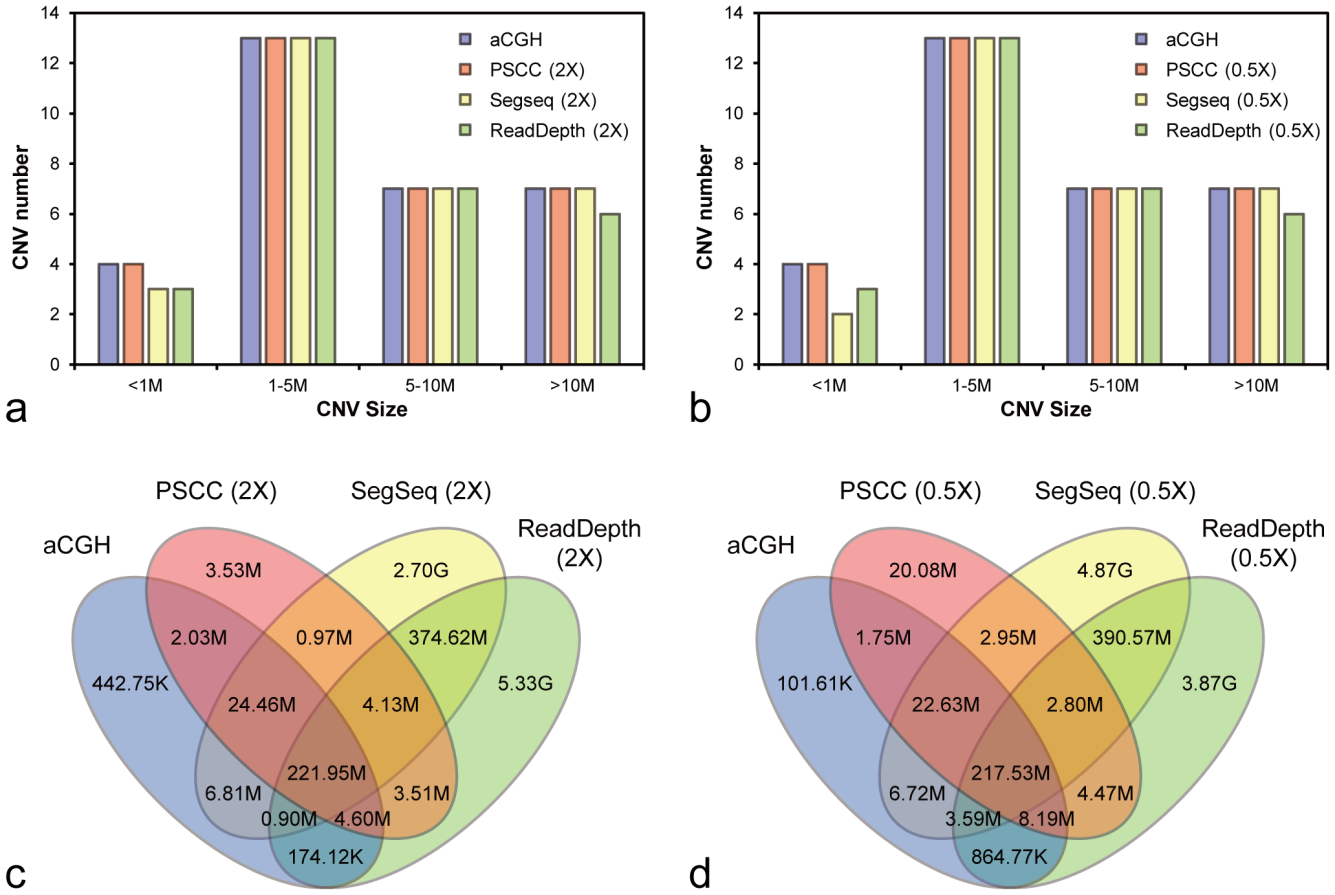
The detection results of confirmed CNVs using PSCC, SegSeq and ReadDepth. The four plots had shown the sensitivity and specificity in three methods with a low coverage sequencing depth. (a) The sensitivity in 2× sequencing; (b) The sensitivity in 0.5× sequencing; (c) The specificity in 2× sequencing; (d) The specificity in 0.5× sequencing.

For sensitivity, we compared the detection rate of the 31 aCGH detected pathogenic CNV between PSCC, SegSeq, and ReadDepth (Figure 4 a). As we mentioned, PSCC successfully detected all these pathogenic CNVs. Also, we found SegSeq accurately detected 34 CNVs but missed one deletion smaller than 1 M, with a total sensitivity of 97.06%. ReadDepth successfully detected 29 CNVs but missed one deletion and one whole chromosome duplication of chrY. The total sensitivity of of ReadDepth is 93.55%.

Meanwhile, we subsampled the clinical samples to 0.5× to estimate the robustness of these methods in ultra-low coverage sequencing (Figure 4 b). All aCGH CNVs were successfully detected in PSCC, whereas SegSeq dropped one deletion and one duplication smaller than 1 Mb. In ReadDepth, one deletion and one whole chromosome Y duplication were still missed. It indicated the good sensitivity of our method in regard to CNV detection of clinical samples, successfully detecting all 31 pathogenic CNVs. Both the result of ∼2× and ∼0.5× sequence depth reveal the advantages of this method when compared to the existing methods.

To compare the specificity of these three methods, we calculated the overlap (cumulated across 34 samples) among these methods (Figure 4 c, d). In this study, aCGH totally detected 261.37 Mbp pathogenic CNVs. For PSCC, 253.04 of 265.19 Mbp (95.42%) CNVs overlapped with the aCGH, whereas only 0.25 of 3.33 Gbp (7.62%) for SegSeq and 0.23 of 5.94 Gbp (3.83%) for ReadDepth. In addition, PSCC, SegSeq and ReadDepth detected 3.53 Mbp, 2.70 Gbp, and 5.33 Gbp method specific CNVs. These numbers indicated the false prediction rate of these three methods would be 1.33% (3.53 Mbp/265.19 Mbp), 81.08% (2.70 Gbp/3.33 Gbp) and 89.73% (5.33 Gbp/5.94 Gbp), implying our correction and statistics strategy can effectively improve the specificity. In the ∼0.5× subsampled set, the situation was almost the same, our method had the lowest false prediction rate of 7.16% (20.07 Mbp/280.39 Mbp), while the SegSeq's increased to 88.22% (4.87 Gbp/5.52 Gbp) and ReadDepth's decreased to 86.08% (3.87 Gbp/4.50 Gbp). It indicated the robustness of our method on false positive control through low sequence coverage. To sum up, our method successfully detects pathogenic CNVs with high sensitivity and specificity, showing robustness and advantages to existing methods, providing promising prospect for clinical application.

## Discussion

In this study, we constructed an efficient bioinformatics method for CNV analysis using an integration process. Large-scale simulation data indicates that our method achieved 99.7% sensitivity and 100% specificity for 300 kb CNVs in a low coverage sequencing condition (i.e. about 2× coverage). For clinical samples, our method effectively detected the CNVs in 34 DC samples (24 Microdeletion/Microduplication syndrome and 10 samples with no obvious pathogenic CNV) using 2× sequence data and 0.5× simulated data, showing great potential for clinical application.

Since only a low sequencing depth is needed, the cost of our method can be controlled at an appropriate level, which is one of the key aspects of large-scale practical applications. Currently, it costs approximately $41 to generate 1 GB of sequence reads [32]. For example, it costs $246 to perform a ∼2× sequencing, which is comparable to the costs of high-resolution aCGH. Moreover, the development of sequencing platforms with lower costs and shorter turnaround times will significantly broaden the application of sequence-based approaches for CNV detection. Our new PSCC method has significant advantages when compared to other bioinformatics methods. We identified all the pathogenic CNVs in the clinical samples, and had much lower false prediction rate compared to other methods. Considering the sensitivity and specificity of our method, it may be a promising solution for basic research and clinical application, both in newborn screening and CNV detection.

There are still some issues that could be improved in the primary stage. Duo to a limitation of the normal control set used in this study, some low frequency polymorphism regions were also retained in the final result. The accumulation of a larger polymorphism database is necessary. Moreover, exogenous gene insertion, small deletions and balanced translocations cannot be detected using this method. The PSCC method should therefore be combined with other methods, such as PEM and *de novo* assembly methods, to make up for these disadvantages. Alongside the progress of the sequencing technologies, especially the advent of the third generation sequencing, we should also consider how the long sequencing read length assisted local assembly can be compositely applied to our method. This strategy not only solves those special structural mutations, but also improves the resolution in the breakpoint boundaries.

## Conclusions

In summary, we have developed a bioinformatics strategy for accurate CNV detection. It broadens the perspective of bias correction and filter strategy of population polymorphisms noise signals in candidate pathogenic CNV detection, and this new PSCC strategy could be a promising new research tool and could assist in the detection of CNVs in a clinical setting.

## Supporting Information

Materials and Methods S1 **Detailed methods and materials that were not listed in the manuscript.**
(DOC)

Table S1 **Statistics of sequence date of all samples mentioned in the paper.**
(XLS)
