# Supplementary material for: PSCC: Sensitive and Reliable Population-Scale Copy Number Variation Detection Method Based on Low Coverage Sequencing
Source: PLoS One. 2014 Jan 21;9(1):e85096. doi: 10.1371/journal.pone.0085096 (PMC3897425; doi:10.1371/journal.pone.0085096)

### Supplementary Material to “PSCC: Sensitive and Reliable Detection of Copy Number Variation based on Population-scale Low Coverage Sequencing”

Xuchao Li, Shengpei Chen, Weiwei Xie, Ida Vogel, Kwong Wai Choy, Fang Chen, Rikke Christensen, Chunlei Zhang, Huijuan Ge, Haojun Jiang, Chang Yu, Fang Huang, Hui Jiang, Xun Xu, Xiuqing Zhang

### Supplementary Methods

#### Sample recruitment

The 90 control samples (CS) were recruited from the CHB and CHS samples in the Pilot 3 study of 1000 Genomes Project. We choose 5X sequencing data for each sample to do the data observation and population-based correction strategy in our study. The 34 Down Syndrome (DS) samples were recruited by the Guangzhou Children’s Social Welfare Home (CHINA). All the DS samples were diagnosed to be typical trisomy 21, each sample was sequenced for ~2X depth. The 34 Danish clinical (DC) samples had received genetic diagnosis by array platform in Department of Clinical Genetics, Aarhus University Hospital (Denmark). Four samples (DC0001 to DC0004) were examined by Bluegnome 4k cytochip, and 30 were analysed using Agilent 180k SurePrint G3 Human CGH microarray. In the arrayCGH analysis, it was found that 24 of them had one or more pathogenic CNVs, including 19 deletions (from 0.4M to 18.3M) and 12 duplications (from 0.4M to 59M), and 10 samples had no obvious pathogenic CNVs (Table S2). Copy number variations (CNVs) in areas containing previously reported CNVs in healthy control samples from the database of genomic variants (DGV) were excluded or assigned as benign. The detailed data production information were listed in “Supplementary Table S1 - DataProduction.xls”.

#### Library construction and sequencing

For massively parallel genomic sequencing, ~200ng of genomic DNA was prepared for library construction. Briefly, gDNA from each sample was sheared by an S2 sonicator (Covaris, Inc), yielding fragments with a predominance of 500 bp in CS and DC, about 800 bp in DS DNA fragments were blunt-ended using T4 DNA polymerase (Enzymatics), Klenow polymerase (Enzymatics), and T4 polynucleotide kinase (Enzymatics) and were ligated to adapters after addition of terminal A nucleotides. The adapter-ligated DNA fragments in the range of 300–350 bp were size-selected by 2% agarose electrophoresis and then underwent 12 cycles of PCR. An Agencourt AMPure 450 ml Kit was used for the purification of PCR products.

The libraries were quality-controlled by Agilent Bioanalyzer DNA 1000 kit (Agilent Technologies) and quantiﬁed by real-time PCR. DNA libraries were hybridized to the surface of sequencing flowcells, and DNA clusters were generated after amplification. The libraries were then sequenced on the Illumina HiSeq^TM^ 2000 sequencing system according to the manufacturer’s instructions.

#### Alignment and basic statistic

All the reads were aligned to the reference genome (HG19, build 37) using SOAP2 with parameters that included inserted DNA size, aligning mismatches and etc. (-l 40 -v 5 -r 1 -s 40 for Hiseq PE100 data, and –v 2 –r 1 for PE50 data). Finally, the average sequence depth of CS were ~5X, DS and DC were ~2X. (Supplementary Table S1 - DataProduction.xls)

After the alignment, we calculated the reads count (RC) and local GC content in each window. The RC was the number of unique mapped reads located in certain window, and we used the middle position of each paired-end reads to represent its location. The local GC content was the mean of the percentage of ‘G’ and ‘C’ in all the reads in each window.

#### Window size estimation

To lessen the influence of the sequence bias to the statistics tests, suitable windows were selected which satisfied the following criteria, (a) read counts in windows should follow *Poisson* distribution well, which was the major premise of most sequenced-based models; (b) windows shared same expected UNIQUE reads count, making it comparable among windows; (c) as small as possible, which could improve resolution.

Therefore, our reference genome was cut into 100-bp sliding simulated reads. Afterward, these simulated reads were mapped to the reference genome with same strategy as sequence data (SOAP2: -l 40 -v 5 -r 1 -s 40), and only unique alignments were retained for following analysis. By setting different windows numbers, expected reads counts and window size were under our control.

Here, we used the RC obtained from DS samples to evaluate the distribution of different copy numbers (CN). (Chromosome X in male was ‘CN=1’, autosomes except 21 was ‘CR=2’ and chromosome 21 was ‘CR=3’) (Figure S1). It showed a series of window sizes that had expected RCs of 25, 50, 100, 150, 250 and 500 in one DS sample (DS-1127). The shared proportions of these three distributions were increasing with the less of expected RCs. So when the window size is divided smaller, the probability of the confused of different copy number is increasing obviously. While the sensitivity and specificity will also increase with the number of window in theory. To balance the performance of detection power and resolution, we chose 150 as theoretical expected RCs in our method.

#### Simulation data generation

In the power estimation, we simulated 66 CNVs (33 duplications and 33 deletions) ranged from 20kb to 2M that located in autosomes. The detailed CNV positions were listed in the table below (Table S3). Simulated sequencing depth was started in an ultra-low coverage 0.2X, and ended in 20X. (0.2, 0.5, 1, 2, 3, 5, 10, 20) Then we randomly generated 100 normal controls based on the CS samples that with the same depth as the test sample. For each depth, we implemented 100 times parallel test to examine the known CNVs.

The GC content correction was executed in each simulated parallel, the population-based normalization was established on the normal controls. Then the binary segmentation and combined test were followed the methods mentioned before (Methods).

#### Sensitivity and specificity statistics

For each simulated CNV, it can be accepted to be detected successfully when the detection proportion is over 60%. Then the sensitivity and specificity of different CNV size were calculated in each sequencing depth.

$$Sensitivity_{size}=\frac{N_{PSCC,true}}{N_{simulate}}$$

$$Specificity_{size}=\frac{N_{PSCC,true}}{N_{PSCC,total}}$$

where $Sensitivity_{size}$and $Specificity_{size}$stands for the sensitivity and specificity for different CNV sizes. $N_{simulate}$means the number of simulated CNV; $N_{PSCC,true}$ means the number of successfully detected CNV by PSCC and $N_{PSCC,total}$means the total number of detected CNV by PSCC;

Finally, the sensitivity and specificity were shown in Figure 3.

#### CNV detection parameters in PSCC, SegSeq and ReadDepth

For the statistic of CNV detected rate in clinical samples, the threshold of detected proportion was also set at 60%. All the 34 clinical samples were accepted PSCC with the 90 CS samples as the normal control set. After that, to compare the detection accuracy between PSCC and other published methods, we did the CNV detection using SegSeq and ReadDepth respectively. The SegSeq were used the suggest parameters of “-W 400 –a 1000 –b 10”, and the control sample used here was a mixture data with ~2X depth which was randomly extracted from CS samples. The parameters of ReadDepth were downloaded from its official website.

At last, to evaluate the performance in ultra-low coverage sequencing, we subsampled the clinical samples to 0.5X to estimate the practice detection power of these methods. We extracted 15 million reads from each sample, and did the detection processes using PSCC, SegSeq and ReadDepth. All the confirmed CNVs founded before can be found in PSCC, while there have 5 and 3 CNVs were lost in SegSeq and ReadDepth (Table S2).

#### Validate of newly detected CNVs

8 duplication regions and 4 deletion regions were selected, which were examined by real-time quantitative PCR (Q-PCR) in 11 DC samples on the ABI StepOne plus Real-Time PCR system (Applied Biosystems) using SYBRGreen Dye. The endogenous control was designed to target the house-keeping gene (GAPDH & HBB) in chromosome 12, avoiding any known structural variations including CNVs.

For data analysis, $C_{t}$values were obtained using Applied Biosystems, input DNA quantities were normalized to house-keeping gene and relative DNA copy number was obtained by pairwise comparisons of test and three control YH DNAs. The $\Delta\Delta C_{t}$ method (Applied Biosystems) was employed to quantify the genomic copy numbers by setting a normal number at two copies. Briefly, a 10uL reaction mixture was made up of 2X concentration SYBR Premix Ex Taq master mix. (TaKaRa).3uM each forward and reverse primers, and 5ng of template DNA. All real-time PCR assays were performed in quadruplicate. Thermal cycling conditions were as follows : 95℃ at 10 min (1cycle) ; 95℃ for 15s, 62℃ for 30s and 72℃ for 30s (40cycles). Melting curves and product validation proceeded per manufacturer’s guidelines. Primers used in this study were ordered from Invitrogen and the sequences were listed in the following (Table S4).

For all CNV regions, the distribution of copy ratio were shown in the figure below (Figure S2), showing that all validate CNVs were truly exist.

### Supplementary Tables

**Table S2. Summary of variation detection in 34 clinical samples**

| **Sample** | **Chr** | **Start** | **End** | **CopyRatio** | **Pathogenic CNVs in Array** | **Benign CNVs in Array** | **qPCR** | **2X** | | **0.5X** | | |
| --- | --- | --- | --- | --- | --- | --- | --- | --- | --- | --- | --- | --- |
|  |  |  |  |  |  |  |  | **SegSeq** | **ReadDepth** | **PSCC** | **SegSeq** | **ReadDepth** |
| DC0001 | 1 | 148,586,648 | 149,812,467 | 0.356 |  |  | √ | √ |  | √ |  |  |
| DC0001 | 16 | 79,705,431 | 88,075,218 | 0.494 | √ |  |  | √ | √ | √ | √ | √ |
| DC0002 | 8 | 59,376,517 | 67,066,979 | 0.494 | √ |  |  | √ | √ | √ | √ | √ |
| DC0003 | 7 | 90,093,918 | 97,521,086 | 0.508 | √ |  |  | √ | √ | √ | √ | √ |
| DC0004 | 15 | 23,533,141 | 28,531,302 | 1.549 | √ |  |  | √ | √ | √ | √ | √ |
| DC0004 | 24 | 2,649,422 | 28,819,361 | 1.944 | √ |  |  | √ |  | √ | √ |  |
| DC0005 | 23 | 77,445,350 | 87,976,237 | 0.517 | √ |  |  | √ | √ | √ | √ | √ |
| DC0006 | 10 | 58,378,758 | 62,257,343 | 0.522 | √ |  |  | √ | √ | √ | √ | √ |
| DC0007 | 1 | 121,086,695 | 121,485,434 | 1.364 |  |  | √ |  | √ | √ |  | √ |
| DC0007 | 5 | 10,379 | 21,516,762 | 0.506 | √ |  |  | √ | √ | √ | √ | √ |
| DC0007 | 11 | 61,303 | 17,924,027 | 1.506 | √ |  |  | √ | √ | √ | √ | √ |
| DC0007 | 12 | 7,827,775 | 8,166,673 | 1.489 |  | √ | √ |  |  | √ |  |  |
| DC0008 | 5 | 105,607,869 | 106,136,623 | 0.527 | √ |  |  | √ | √ | √ | √ | √ |
| DC0008 | 17 | 21,380,581 | 21,947,162 | 1.385 |  | √ | √ | √ | √ | √ |  | √ |
| DC0009 | 1 | 148,511,358 | 148,853,335 | 0.671 |  | √ |  |  | √ | √ |  |  |
| DC0009 | 19 | 12,469,177 | 14,390,812 | 0.542 | √ |  |  | √ | √ | √ | √ | √ |
| DC0010 | 20 | 4,949,136 | 5,354,227 | 1.471 | √ |  |  | √ | √ | √ | √ | √ |
| DC0011 | 1 | 120,493,365 | 120,936,695 | 0.735 |  |  |  |  |  | √ |  |  |
| DC0011 | 17 | 16,718,466 | 20,599,190 | 0.513 | √ |  |  | √ | √ | √ | √ | √ |
| DC0011 | 22 | 23,763,464 | 25,067,214 | 1.473 | √ |  |  | √ | √ | √ | √ | √ |
| DC0012 | 8 | 10,082 | 6,757,602 | 1.533 | √ |  |  | √ | √ | √ | √ | √ |
| DC0012 | 22 | 47,665,197 | 51,244,174 | 0.529 | √ |  |  | √ | √ | √ | √ | √ |
| DC0013 | 18 | 72,844,656 | 75,532,868 | 0.517 | √ |  |  | √ | √ | √ | √ | √ |
| DC0014 | 14 | 101,775,037 | 106,019,765 | 0.542 | √ |  |  | √ | √ | √ | √ | √ |
| DC0015 | 7 | 154,581,177 | 159,128,563 | 0.515 | √ |  |  | √ | √ | √ | √ | √ |
| DC0015 | 13 | 114,151,318 | 115,109,778 | 1.568 | √ |  |  | √ | √ | √ |  | √ |
| DC0015 | 19 | 24,123,629 | 24,485,850 | 1.464 |  | √ | √ |  |  | √ | √ |  |
| DC0016 | 2 | 61,548,640 | 66,253,292 | 1.499 | √ |  |  | √ | √ | √ | √ | √ |
| DC0017 | 9 | 6,604,106 | 7,258,641 | 0.743 | √ |  |  |  |  | √ |  |  |
| DC0017 | 9 | 11,948,602 | 12,286,521 | 0.53 |  | √ | √ | √ | √ | √ | √ | √ |
| DC0018 | 12 | 71,168,232 | 77,337,389 | 0.508 | √ |  |  | √ | √ | √ | √ | √ |
| DC0019 | 11 | 174,618 | 8,208,052 | 1.509 | √ |  |  | √ | √ | √ | √ | √ |
| DC0019 | 17 | 1 | 1,705,738 | 0.533 | √ |  |  | √ | √ | √ | √ | √ |
| DC0020 | 4 | 189,383,537 | 189,716,809 | 1.543 |  | √ | √ |  | √ | √ | √ | √ |
| DC0021 | 7 | 64,590,167 | 65,166,471 | 0.514 |  | √ | √ |  | √ | √ |  |  |
| DC0023 | 6 | 113,232,530 | 113,589,479 | 0.665 |  | √ | √ |  |  | √ |  |  |
| DC0028 | 10 | 94,145,218 | 94,468,676 | 1.469 |  | √ | √ |  | √ | √ |  | √ |
| DC0029 | 15 | 23,517,633 | 28,536,910 | 1.543 | √ |  |  | √ | √ | √ | √ | √ |
| DC0029 | 21 | 47,398,326 | 47,838,323 | 1.541 |  | √ | √ | √ | √ | √ |  |  |
| DC0031 | 4 | 75,790 | 18,815,443 | 0.498 | √ |  |  | √ | √ | √ | √ | √ |
| DC0031 | 4 | 18,815,443 | 48,309,196 | 1.508 | √ |  |  | √ | √ | √ | √ | √ |
| DC0032 | 8 | 138,633,668 | 139,304,225 | 1.419 |  | √ | √ | √ | √ | √ | √ | √ |
| DC0032 | 9 | 218,079 | 38,795,151 | 1.483 | √ |  |  | √ | √ | √ | √ | √ |
| DC0032 | 23 | 8,410,847 | 9,081,802 | 1.533 |  | √ |  | √ | √ | √ | √ | √ |
| DC0033 | 15 | 30,439,758 | 32,894,361 | 0.465 | √ |  |  | √ | √ | √ | √ | √ |
| DC0033 | 16 | 34,173,150 | 34,830,111 | 1.272 |  | √ |  | √ |  | √ |  |  |
| DC0034 | 7 | 45,044,457 | 47,396,858 | 0.506 | √ |  |  | √ | √ | √ | √ | √ |

The “√” means successfully detected in the process of array, qPCR or bioinformatics methods.

**Table S3. The simulated CNV positions and copy ratio**

| **Chr** | **Start** | **End** | **Copy Ratio** | **Chr** | **Start** | **End** | **Copy Ratio** |
| --- | --- | --- | --- | --- | --- | --- | --- |
| 1 | 8,200,453 | 10,200,452 | 1.5 | 10 | 77,328,885 | 77,348,884 | 0.5 |
| 1 | 18,397,753 | 18,497,752 | 0.5 | 10 | 130,291,711 | 130,341,710 | 1.5 |
| 1 | 32,421,152 | 32,441,151 | 1.5 | 12 | 14,576,145 | 14,606,144 | 0.5 |
| 1 | 45,034,414 | 45,109,413 | 0.5 | 12 | 50,829,852 | 50,849,851 | 0.5 |
| 1 | 54,828,711 | 55,328,710 | 1.5 | 12 | 54,137,590 | 54,167,589 | 1.5 |
| 1 | 62,325,529 | 62,625,528 | 0.5 | 12 | 100,087,665 | 100,387,664 | 1.5 |
| 1 | 107,561,413 | 107,636,412 | 0.5 | 12 | 116,772,255 | 116,822,254 | 1.5 |
| 2 | 6,510,405 | 7,010,404 | 0.5 | 12 | 120,115,750 | 120,865,749 | 1.5 |
| 2 | 26,104,231 | 26,604,230 | 0.5 | 13 | 28,393,159 | 28,893,158 | 1.5 |
| 2 | 104,544,348 | 104,619,347 | 1.5 | 13 | 62,429,420 | 63,179,419 | 1.5 |
| 2 | 203,604,668 | 204,104,667 | 1.5 | 14 | 69,662,517 | 69,862,516 | 0.5 |
| 2 | 224,850,215 | 224,950,214 | 1.5 | 14 | 76,528,458 | 78,528,457 | 0.5 |
| 3 | 25,320,818 | 25,340,817 | 0.5 | 14 | 95,116,223 | 96,116,222 | 1.5 |
| 3 | 69,194,989 | 69,269,988 | 1.5 | 14 | 98,342,873 | 100,342,872 | 0.5 |
| 3 | 71,409,405 | 71,709,404 | 1.5 | 15 | 65,658,191 | 65,708,190 | 1.5 |
| 3 | 172,513,188 | 172,813,187 | 0.5 | 15 | 95,332,936 | 96,332,935 | 1.5 |
| 4 | 24,702,702 | 24,902,701 | 1.5 | 16 | 6,856,591 | 7,056,590 | 0.5 |
| 4 | 116,092,876 | 116,122,875 | 0.5 | 16 | 13,647,099 | 13,722,098 | 1.5 |
| 4 | 154,923,646 | 155,123,645 | 1.5 | 16 | 50,239,014 | 50,339,013 | 0.5 |
| 5 | 5,694,584 | 5,894,583 | 0.5 | 16 | 66,727,958 | 66,747,957 | 1.5 |
| 5 | 10,741,834 | 11,041,833 | 1.5 | 16 | 78,108,893 | 78,608,892 | 0.5 |
| 5 | 171,820,786 | 171,840,785 | 1.5 | 17 | 8,495,414 | 8,525,413 | 1.5 |
| 6 | 36,988,943 | 37,738,942 | 0.5 | 17 | 38,118,867 | 38,168,866 | 0.5 |
| 6 | 97,485,559 | 97,785,558 | 0.5 | 17 | 48,515,907 | 48,545,906 | 0.5 |
| 7 | 41,448,700 | 41,548,699 | 1.5 | 17 | 55,591,688 | 56,341,687 | 1.5 |
| 7 | 134,989,148 | 135,039,147 | 0.5 | 17 | 71,581,449 | 73,581,448 | 0.5 |
| 7 | 138,197,020 | 138,297,019 | 0.5 | 19 | 10,146,982 | 10,896,981 | 0.5 |
| 8 | 3,547,408 | 3,647,407 | 1.5 | 19 | 17,151,629 | 19,151,628 | 1.5 |
| 8 | 38,431,771 | 39,181,770 | 0.5 | 20 | 49,805,443 | 50,805,442 | 0.5 |
| 8 | 53,471,695 | 53,501,694 | 1.5 | 20 | 55,896,902 | 56,896,901 | 0.5 |
| 9 | 11,201,878 | 11,276,877 | 0.5 | 21 | 38,036,130 | 39,036,129 | 1.5 |
| 9 | 130,916,095 | 131,916,094 | 0.5 | 22 | 27,051,204 | 27,101,203 | 0.5 |
| 10 | 70,502,262 | 70,702,261 | 1.5 | 22 | 44,190,136 | 46,190,135 | 1.5 |

**Table S4. Primer design for CNV validation**

| **ID** | **Sample** | **Chr** | **Start** | **End** | **CopyRatio** | **Forward primer** | **Reverse primer** |
| --- | --- | --- | --- | --- | --- | --- | --- |
| No.01 | DC0001 | 1 | 148,586,648 | 149,812,467 | 0.36 | GCATGAGCCCCAAAAACTGGAGC | CCGGTCTAGCCCACATCACTCCC |
| No.02 | DC0007 | 1 | 121,086,695 | 121,485,434 | 1.36 | ACCCTGGAACTGGTGCCGCTC | CCACACACCCCACTGCCGTCT |
| No.03 | DC0007 | 12 | 7,827,775 | 8,166,673 | 1.49 | GGTTGCAGTGACCCGAAATGGC | TGATGCCCGGCCTGTTGTTTCT |
| No.04 | DC0008 | 17 | 21,380,581 | 21,947,162 | 1.39 | CACGACAGGGCTTGAGCATCCA | ATGTGCTGGCGCTGTGTTCCTG |
| No.05 | DC0015 | 19 | 24,123,629 | 24,485,850 | 1.46 | GAGAGAGGGGAGGGGGCTGGTT | TCAGCGCAGCCGCCATCTTATT |
| No.06 | DC0017 | 9 | 11,948,602 | 12,286,521 | 0.53 | GTGGCCACAGGGGTCCAGTCA | TGCCCGGGGGAATTCTCTCAA |
| No.07 | DC0020 | 4 | 189,383,537 | 189,716,809 | 1.54 | TTCTCAAACGCCCAGGCACCAT | GAGGGAGGGAGGCGGAAACAGA |
| No.08 | DC0021 | 7 | 64,590,167 | 65,166,471 | 0.51 | TCACCCCAGCGTGGGCTAAAGA | GCCCTCCATCCAGGGTGACAGA |
| No.09 | DC0023 | 6 | 113,232,530 | 113,589,479 | 0.67 | TCCACAGGAACCAGAGCCAGGG | AGTGCATGCCCCACCTTCCGTA |
| No.10 | DC0028 | 10 | 94,145,218 | 94,468,676 | 1.47 | ACCGTGCCCAGCCAACTTAGCA | CCCTACTGCTTTGCGTGCCACC |
| No.11 | DC0029 | 21 | 47,398,326 | 47,838,323 | 1.54 | GCACCCACCTGGGCATGAGACT | AGCACCCAGCCCATCACCACTC |
| No.12 | DC0032 | 8 | 138,633,668 | 139,304,225 | 1.42 | CCAGCTCCTGGTGTGGCATCCT | GGACAGGGGAGCACCCACTTGA |

### Supplementary Figures

**Figure S1. The RC distribution of copy numbers in different bin sizes that contained various expected RCs**

(a)-(f) stand for the copy number distribution in different bin size that had the expected RCs of 25, 50, 100, 150, 250 and 500 respectively. The very light gray means copy number of 1 (chromosome X in male samples), light gray means copy number of 2 (autosomes) and gray means copy number of 3 (chromosome 21 in DS samples).


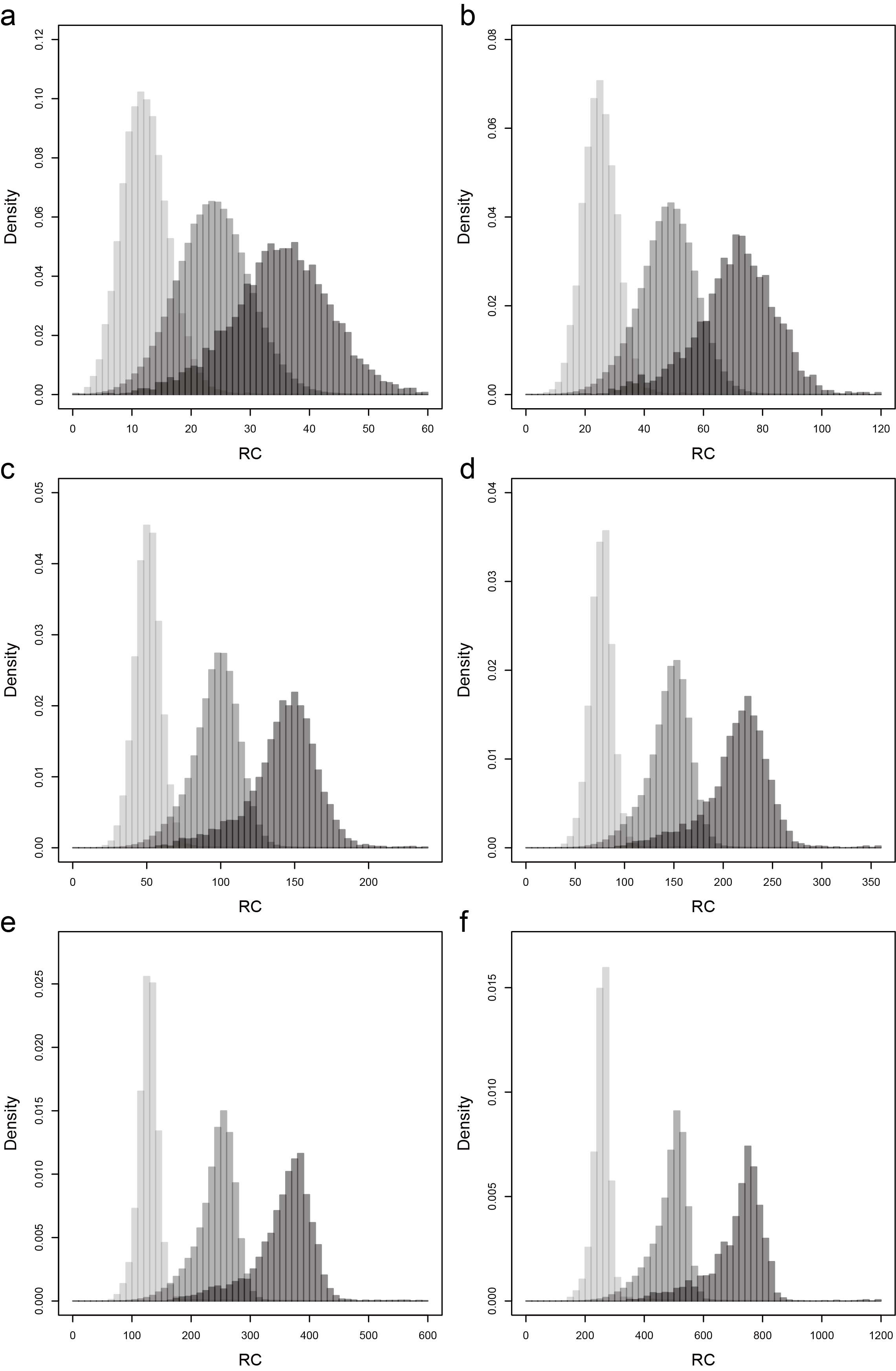


**Figure S2. qPCR validate result of newly detected CNVs**

12 novel detected CNVs were validated by qPCR with the YH sample as a normal control. These CNVs were located in 11 different samples, contained 4 deletion and 8 duplications. The colors stands for 3 parallel experiments, Y-axis means the copy ratio obtained in qPCR experiment.


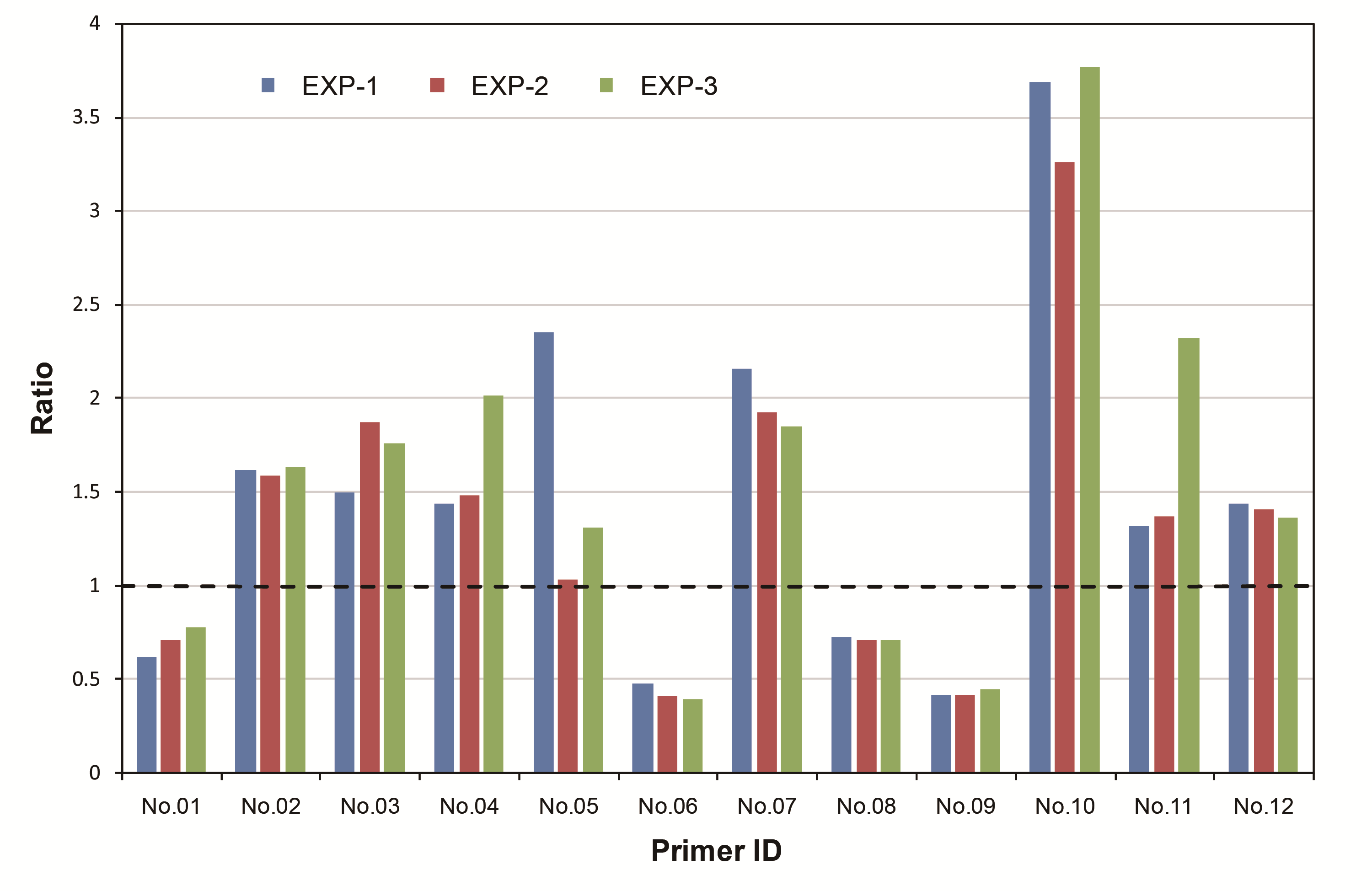

Supplement: Materials and Methods S1 — Detailed methods and materials that were not listed in the manuscript. (DOC) [file pone.0085096.s001.doc]
